# MiR-195-5p is a Potential Factor Responsible for CPNE1 Differential Expression between Subtypes of Non-Small Cell Lung Cancer

**DOI:** 10.7150/jca.39884

**Published:** 2020-02-19

**Authors:** Wenwen Du, Ting Liu, Yang Zhang, Yuanyuan Zeng, Jianjie Zhu, Haicheng Tang, Zeyi Liu, Jian-an Huang

**Affiliations:** 1 Department of Respiratory Medicine, the First Affiliated Hospital of Soochow University, Suzhou, 215006, China;; 2 Suzhou Key Laboratory for Respiratory Diseases, Suzhou, 215006, China;; 3 Institute of Respiratory Diseases, Soochow University, Suzhou, 215006, China;; 4 Department of Respiratory Medicine, the First People's Hospital of Yancheng City, Yancheng, 224001, China.

**Keywords:** CPNE1, non-small cell lung cancer, tumor subtype, miR-195-5p, differential expression

## Abstract

**Purpose:** Lung cancer is the most common malignancy with poor 5-year survival among men and women. Previous studies have shown that CPNE1 is up-regulated in non-small cell lung cancer (NSCLC). However, whether and how CPNE1 expression varies between different subtypes of NSCLC remains less understood.

**Methods:** Bioinformatical analysis and GSE19188 were selected to confirm CPNE1 expression in different subtypes of NSCLC. Four microRNA prediction websites and GSE53883, GSE43000 were used to evaluate the possible targeting microRNAs. Kaplan-Meier survival curves were drawn based on Tumor Lung Bild -114 dataset using R2, UCSC Xena browser or linkedomics platform. Furthermore, we verified our prediction via qRT-PCR, and western blot and luciferase reporter assays.

**Results:** we demonstrated that higher CPNE1 expression was associated with poorer survival in NSCLC patients. Moreover, among the different subtypes, patients with squamous cell lung cancer (SCC) exhibited higher level of CPNE1 expression, as well as substantially poorer survival. MiR-195-5p was down-regulated in NSCLC tissues. Interestingly, SCC patients showed lower miR-195-5p expression compared to patients with lung adenocarcinoma (ADC). In addition, functional assays proved that miR-195-5p overexpression inhibited the proliferation, migration, and invasion of NSCLC-derived cells by directly targeting CPNE1. Pathway analysis showed decreased expression of p-AKT, p-Erk, and Snail after transfection with miR-195-5p mimics in both lung adenocarcinoma and squamous cell lines.

**Conclusion:** Our findings suggested that miR-195-5p regulation contributed to the differential expression of CPNE1 in NSCLC subtypes.

## Introduction

Lung cancer is the dominant cause of cancer deaths in men and women worldwide 1. Approximately 85% of all cases belong to non-small cell lung cancer (NSCLC), which mainly includes lung adenocarcinoma (ADC) and squamous cell lung cancer (SCC) 2, 3. Molecular target therapy and clinical immunotherapy have allowed for remarkable therapeutic improvement and contributed to prolong the survival of patients with advanced NSCLC. However, the 5-year survival rate remains approximately 10% 4, 5. Thus, new molecular targets for NSCLC need to be identified.

The CPNE gene family includes nine members encoding calcium-dependent phospholipid-binding proteins (copine 1-9 in humans), which are evolutionary conserved from plants to Homo sapiens 6, 7. Copine 1 (CPNE1) is expressed in various tissues and organs, including lung tissues 8. Similar to other members of the family, CPNE1 is composed of two N-terminal C2 domains (C2A and C2B), involved in cell signaling and membrane trafficking pathways, and an A domain at the C-terminus, which is reported to interact with intracellular proteins 9, 10. Various studies have proven that CPNE1 plays a calcium-independent role in neuronal differentiation via AKT phosphorylation 11, 12. Moreover, it can interact with partner proteins, such as 14-3-3γ and JAB1, to induce intracellular signal transduction regulating the functions of neuronal stem cells 13, 14. In addition to functioning in neuronal differentiation, CPNE1, along with Annexin A1 and Annexin A5, can also regulate the process of autophagosome maturation in Ca2+-dependent manner 15, 16.

Accumulating evidence indicates a key role of CPNE1 in cancer progression and metastasis. CPNE1 is highly expressed in prostate cancer and its expression is positively associated with TRAF2 expression, and is related to advanced tumor stages and poor survival 17. In addition, CPNE1 silencing can inhibit osteosarcoma cell proliferation, invasion, and migration, suggesting a potential target for osteosarcoma therapy 18. In NSCLC, our previous studies demonstrated that CPNE1 played a vital role in the regulation of cell growth, migration and invasion, serving as a downstream target of miR-335-5p 19, 20.

Owing to the above mentioned studies, we know that CPNE1 is aberrantly up-regulated in NSCLC tissues. However, whether CPNE1 expression varied in different subtypes of NSCLC remained unclear. Based on the public database, we know that CPNE1 expression is higher in SCC subtype when compared to ADC tissues in lung cancer. So the next step is to verify the finding in our own NSCLC tissues so that we can see if these observed differences are actually real. Moreover, the underlying mechanism may also need to be further explored.

MicroRNAs are essential epigenetic regulators modifying the expression and function of target genes 21, 22. The microRNA-mRNA network is an established regulatory system influencing gene expression at the post-transcriptional level 23, 24. After integrated analysis with public database, it is surprisingly to find that miR-195-5p expression is lower in SCC subtype when compared to ADC tissues. MiR-195-5p is characterized as a tumor suppressor in cancer development 25-27. However limited studies have demonstrated the role of miR-195-5p in lung cancer. It is reported that miR-195-5p had lower expression in lung cancer and may act as a biomarker that contributed to the diagnosis of lung cancer and the detection of its high-risk population 28, 29. Moreover, miR-195-5p functions as a predictor of poor prognosis by directly targeting CIAPIN1 30. In addition, miR-195-5p Inhibited NSCLC cell proliferation and induced apoptosis by targeting CEP55^31^. So we hypothesized whether miR-195-5p expression may be responsible for the differential expression among subtypes of lung cancer.

Our present study is the first to demonstrate differential expression of CPNE1 in ADC and SCC subtypes and identify that microRNA-195-5p may be responsible for the regulatory mechanisms. These findings may contribute to the design of tumor subtype-specific therapeutic strategies.

## Material and Methods

### Tissue samples

Fifty-six NSCLC tissues and paired noncancerous lung tissues were collected from patients between 2012 and 2016 at the respiratory department of First People's Hospital of Soochow University. At recruitment, all participants signed a written informed consent. All patients were 18-80 years old with Eastern Cooperative Oncology Group (ECOG) score of 0-2. Besides, patients were histologically and pathologically diagnosed according to the Revised International System for Staging Lung Cancer after surgery. All patients have not undergone chemotherapy or radiotherapy prior to tissue sampling. And the corresponding non-cancerous lung tissues were 5cm distance from the cancer margin. More importantly, patients who had received chemotherapy or radiotherapy before tissue sampling or those who were diagnosed with small cell lung cancer or combined with other kind of cancers were excluded from our study. Patients diagnosed with other systemic infections or with severe heart, brain, liver and kidney diseases were also not involved. The tissue samples were stored frozen at -80°C. This study was approved by the Ethics Committee of the First People's Hospital of Soochow University.

### Prediction of the association between CPNE1 expression and overall survival in NSCLC patients

The R2 web-based application (http://r2.amc.nl) was used to generate Kaplan-Meier survival curves between CPNE1 expression and overall survival (OS; 2, 5, and 10 years) of NSCLC patients in the Tumor Lung Bild -114 dataset. Kaplan-Meier curves were generated using the auto-select best cutoff value. We also evaluated the association in the Kaplan-Meier Plotter database(http://kmplot.com/) to further clarify the impact of CPNE1 expression on patients' OS.

### Correlation analysis between CPNE1 expression in NSCLC subtypes and overall survival

We applied the Oncomine database (https://www.oncomine.org) and the GEO datasets GSE19188 to retrieve data on CPNE1 expression in NSCLC and normal control tissues (adjusted p<0.05, absolute log2 fold change >2). In addition, CPNE1 expression was deeply analyzed in histologically distinct lung adenocarcinomas and squamous cell lung cancers. To validate the hypothesis that differential CPNE1 expression in various NSCLC subtypes could result in different OS, we extracted data from the TCGA lung dataset using the UCSC Xena browser (http://xenabrowser.net).

### CPNE1 -associated microRNAs

Four relevant databases for predicting microRNA-mRNA interactions were used to identify possible CPNE1-targeting microRNAs: Targetscan (http://www.targetscan.org/), StarBasev2.0 (http://starbase.sysu.edu.cn/starbase2), miRDB (http://www.mirdb.org/), and RNAhybrid (http://bibiserv.techfak.unibielefeld.de/rnahybrid/). All candidate microRNAs were compared by the four databases. Finally, a Venn diagram was built and 17 common microRNAs were identified.

### MicroRNA expression in NSCLC subtypes and overall survival correlation analysis

Candidate microRNA expression was analyzed in the GSE53882 and GSE43000 datasets. GSE53882 is a public dataset that contains data of 397 NSCLC patients and 151 corresponding adjacent noncancerous tissues. We selected prognostic microRNAs with differential expression between these two groups. GSE4300 is another dataset that contains information on transcriptional microRNA differences between squamous cell carcinoma and adenocarcinoma. The functions and associations of microRNAs with overall survival were further validated using the online database Linkedomics (http://www.linkedomics.org). The threshold for statistical significance was set at *P*<0.05.

### Cell culture

The NSCLC cell lines A549, H1299, H226, and SK-MES-1 were obtained from the Cell Bank of the Chinese Academy of Sciences (Shanghai, China) and cultured in RPMI‑1640 and MEM medium (Hyclone, South Logan, UT, USA) supplemented with 10% fetal bovine serum (Gibco, Carlsbad, CA, USA) at 37˚C in a humidified incubator containing 5% CO2.

### RNA extraction and quantitative real-time PCR analysis

RNA isolation, cDNA synthesis, and quantitative real-time PCR analysis were performed as previously described 32. The primer sequences used for CPNE1 mRNA detection were as follows: 5′-ACCCACTCTGCGTCCTT-3′ (forward primer) and 5′-TGGCGTCTTGTTGTCTATG-3′ (reverse primer), and those used for β-actin mRNA detection were as follows: 5'-CACAGAGCCTCGCCTTTGCC-3' (forward primer) and 5'-ACCCATGCCCACCATCACG -3' (reverse primer). The primers for miR-195-5p were purchased from RiboBio Co. Ltd (Guangzhou, China). β-actin and U6 were served as internal controls. The △△Ct method was applied to calculate the relative expression of these mRNAs.

### Plasmid construction, transient transfection, and luciferase assay

The predicted CPNE1 3'-UTR fragments containing miR-195-5p target (positions 134-140) and mutant binding sites were synthesized ex novo (Genewiz, Suzhou, China) and fused to the 3'-end of the psi-CHECK2 dual luciferase reporter vector (Promega, Madison, WI, USA). Cells were plated in a 24-well plate and co-transfected with the constructed plasmids with either miR-195-5p mimics or miR-NC using Lipofectamine 2000 (Life Technologies, Carlsbad, CA, USA). Then 24-48h later, the cell lysate was collected and luciferase activity was measured using a Dual-Luciferase Reporter Assay kit (Promega). Each experiment was performed in triplicate.

### Cell growth and clonogenic assay

Cell proliferation was examined using Cell Counting Kit-8 (Boster, Wuhan, China). Briefly, after transfection with miR-195-5p and miR-NC mimics, 3000 cells were seeded into 96-well plates. CCK-8 reagent was added to each well after 24 h, 48 h, and 72 h. Cell viability was determined by measuring the absorbance at 450 nm and 630 nm. For clonogenic assays, cells were cultured for 7-10 days until foci formation. Then, the cells were stained with Giemsa and counted. The experiment was performed in triplicate.

### Migration and invasion assay

Cell migration and invasion assays were performed using transwell chambers (Corning, New York, NY, USA). The only difference between these two assays was that, for the invasion assay, the inserts were pre-coated with Matrigel matrix (BD Science, Sparks, MD, USA) at 37˚C for 2 h. In both assays, after transfection with miR-195-5p mimics or miR-NC for 48 h, 4×10^4^ cells were suspended per well and diluted in 200 μl medium containing 1% FBS. Next, cells were added to the upper chamber and 800 μl medium containing 10% FBS were added into each bottom chamber. Twenty-four hours later, cells that had migrated through the insert were fixed with methanol for 30 min, air-dried for 10 min, stained with 0.1% crystal violet overnight, and washed thrice with PBS. Cells were photographed and counted. Each experiment was performed in triplicate.

### Western blot assay

Cells were lysed in RIPA buffer (Cell Signaling Technology, Danvers, MA, USA) supplemented with protease inhibitor and phosphatase inhibitor cocktail (Sigma-Aldrich, St. Louis, MO, USA). Proteins were separated by 10% SDS-PAGE and transferred to nitrocellulose membranes (Millipore, Billerica, MA, USA). Immunoblots were blocked with 5% BSA for 1 h at room temperature and then incubated with specific primary antibodies overnight at 4°C, followed by appropriate secondary antibodies. Then, after washing four times with TBST, chemiluminescence was used for detection (Pierce, Rockford, IL, USA). The antibodies involved were anti-CPNE1 (Z6, Santa Cruz Biotechnology), anti-p-AKT (Ser473), anti-AKT (D9E), anti-p-Erk (Thr202/Tyr202), anti-Erk (137F5), and anti-Snail (C15D3) (Cell Signaling Technology, Danvers, MA, USA).

### Statistical analysis

All results obtained are presented as the mean ± standard deviation (SD). Statistical significance was analyzed with Student's t‑test and *P*<0.05 was regarded as significant. All statistical analyses were performed using GraphPad Prism 5.0 (GraphPad, San Diego, CA, USA) and SPSS 7.0 software (SPSS, Chicago, IL, USA).

## Results

### Aberrantly up-regulated CPNE1 expression might predict poor OS in NSCLC patents

**Figure 1:**
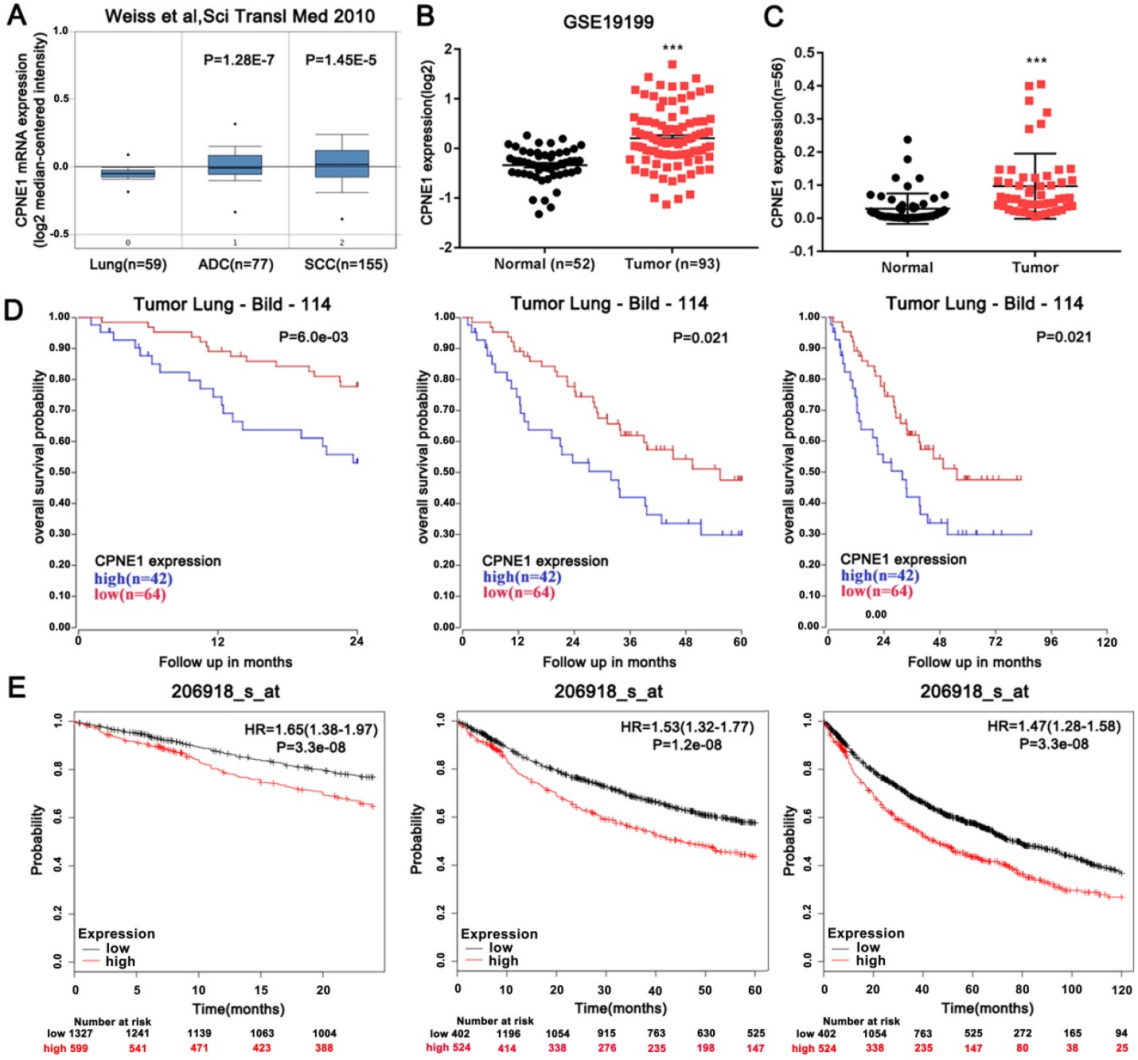
** Aberrantly expressed CPNE1 might predict reduced OS in NSCLC patients.** (A-C) CPNE1 expression is upregulated according to the public Oncomine dataset GSE19188, and our 56 paired tissues. (D-E) Kaplan-Meier curves showed that higher expression of CPNE1 is associated with poorer 2-, 5-, and 10-year survival. *P<0.05, ***P<0.001.

The expression level of CPNE1 was examined using the publicly available Oncomine database. Weiss reported that CPNE1 is highly expressed in both ADC and SCC tumors, when compared to normal lung tissues (Figure 1A). From NCBI-GEO, a public platform for gene or microarray expression profile, we selected the GSE19188 dataset, which included data of 52 normal lung tissues and 93 NSCLC tissues. Similarly up-regulated CPNE1 expression was found in NSCLC tissues (Figure 1B). To validate this result, 56 paired NSCLC tissues and corresponding non-cancerous lung tissues were examined by qRT-PCR analysis, revealing increased CPNE1 expression in NSCLC tissues (Figure 1C). Based on data extracted from the R2 database, we further explored the association between CPNE1 expression and the 2-, 5-, and 10-year overall survival (OS) rates in NSCLC patients. Higher CPNE1 expression was associated with poorer survival rate at all follow-ups (Figure 1D). Kaplan-Meier Plotter database also showed that up-regulation of CPNE1 expression was indicative of poor survival, which was consistent with previous findings (Figure 1E).

### Comparison of CPNE1 expression between different NSCLC subtypes

**Figure 2:**
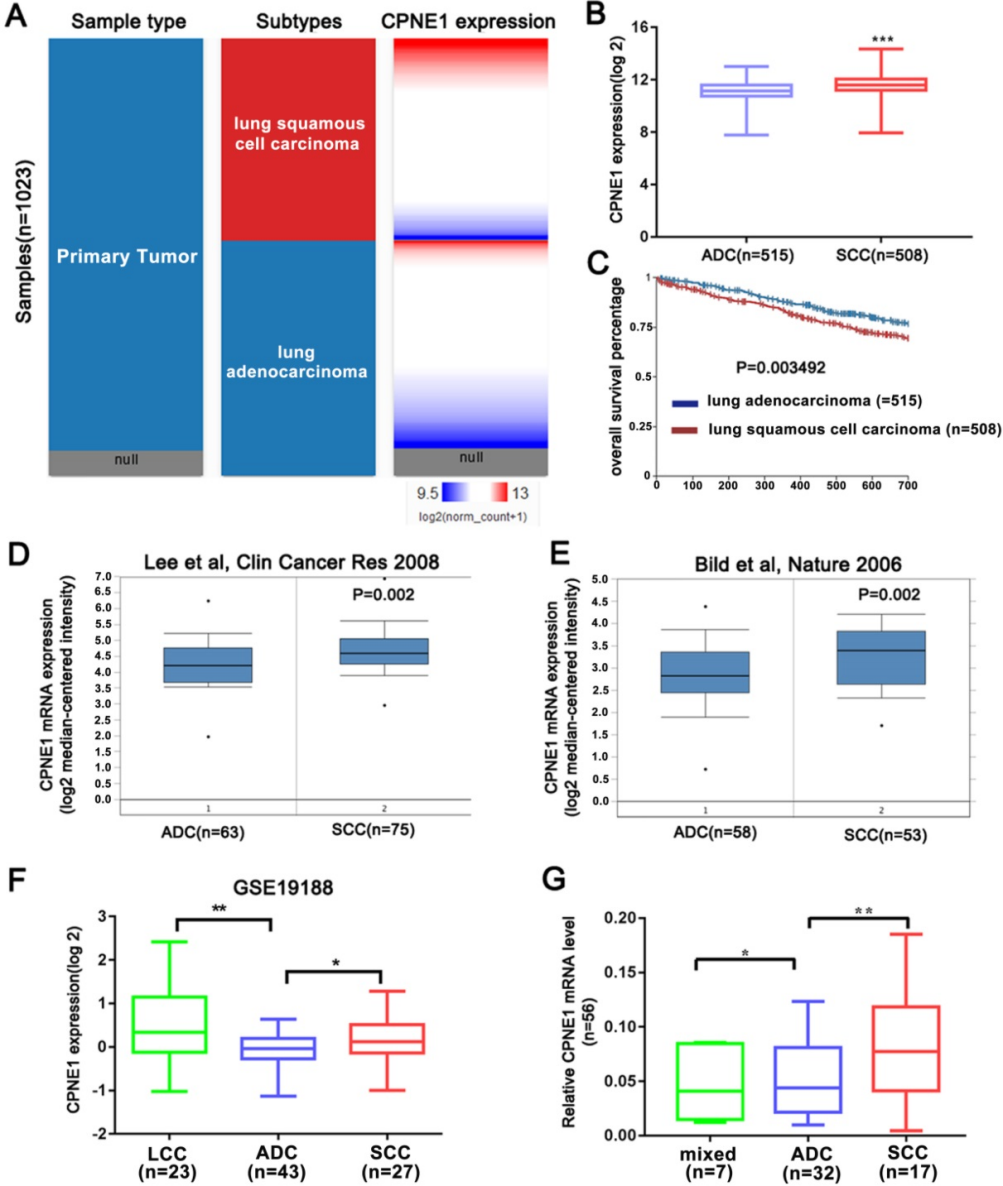
** Further analysis of CPNE1 expression in different NSCLC subtypes.** (A-B) The heat map and box plots showed higher CPNE1 expression in SCC compared to ADC subtype. (C) Kaplan-Meier survival curves showed poorer prognosis in SCC subtypes. (D-F) SCC tumors contained higher CPNE1 expression compared to the ADC tumors, according to Oncomine and GSE19188 datasets. (G) Differential CPNE1 expression in NSCLC subtypes was further confirmed in 56 paired tissues. *P<0.05, **P<0.01, and ***P<0.001.

The above results indicated that CPNE1 was highly expressed in NSCLC. However whether CPNE1 expression varied in different subtypes of NSCLC remained unknown. Based on information retrieved from the Oncomine database, the Bild and Lee datasets demonstrated that CPNE1 expression is higher in the SCC compared to the ADC subtype (Figure 2D, E). CPNE1 expression differs in large cell carcinoma (LCC), ADC, and SCC subtypes in the GSE19188 dataset, and we observed differential expression between ADC and SCC tumors (Figure 2F). Moreover, we analyzed additional tissue samples, which included 30 ADC, 17 SCC, and 7 mixed NSCLC subtypes. CPNE1 expression in the SCC subtype was significantly higher compared to the ADC subtype (Figure 2G). As shown in Figure 2A and 2B, a heat map and the corresponding box plots confirmed that SCC tumors exhibited higher CPNE1 expression. Kaplan-Meier survival curves showed a significant difference in the 2-year survival between ADC and SCC subtypes. In particular, the patients with the ADC subtype exhibited higher 2-year survival rate (Figure 2C).

### Candidate CPNE1-related microRNAs

**Figure 3:**
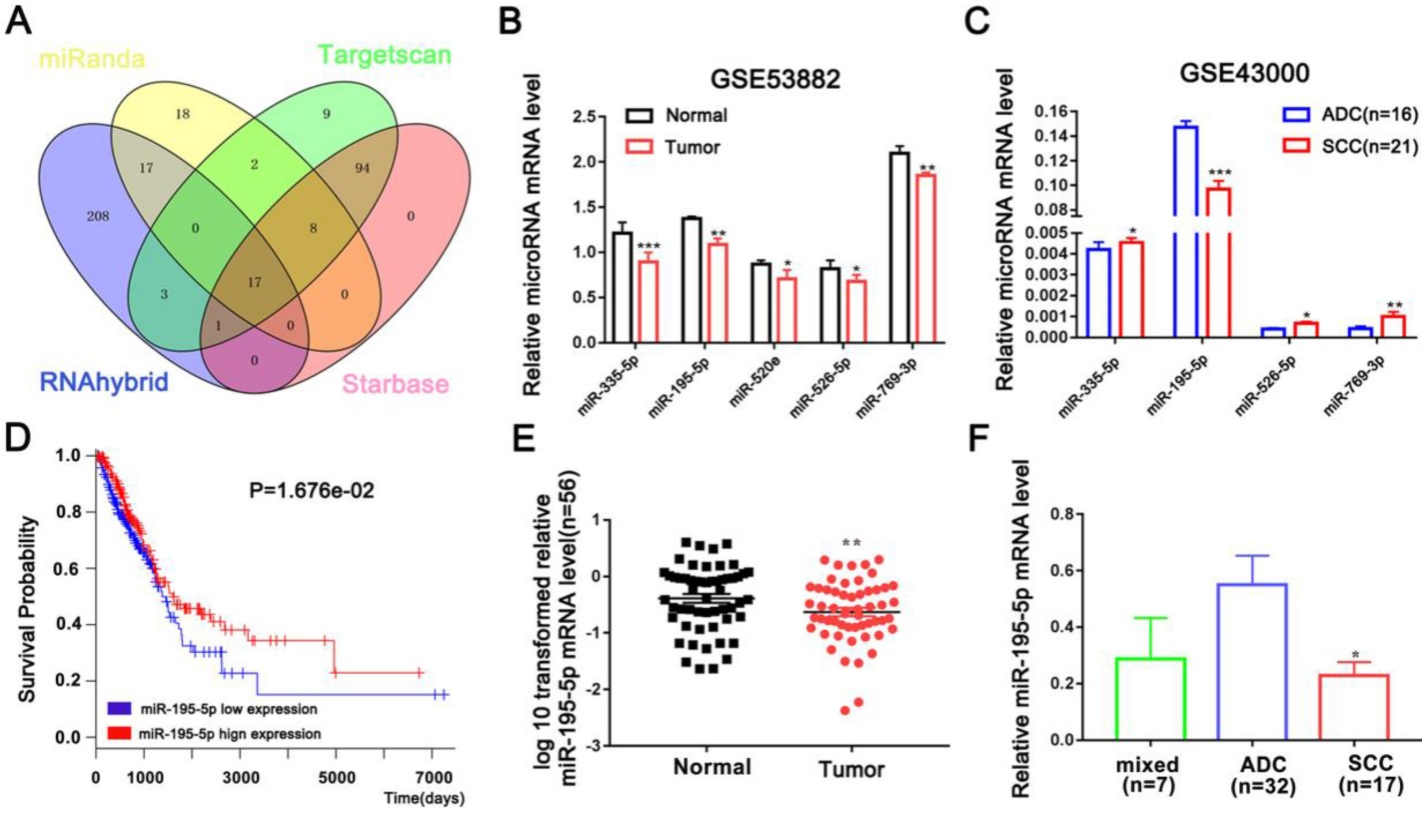
** Candidate CPNE1-related microRNAs.** (A) Seventeen microRNAs were identified by all four bioinformatics datasets. (B-C) The relative expression of downregulated microRNAs was verified in the GSE53882 and GSE4300 datasets. Among the five predicted microRNAs, only miR-195-5p expression was lower in the SCC compared to the ADC subtype. (D) Kaplan-Meier survival curves indicated that higher miR-195-5p expression was associated with better prognosis. (E) MiR-195-5p expression was downregulated in 56 paired NSCLC tissues. (F) MiR-195-5p expression was higher in the ADC than the SCC subtype, contrary to CPNE1 expression. *P<0.05, **P<0.01, and ***P<0.001.

A series of microRNAs that can potentially target CPNE1 were obtained from four public databases. Among all candidates, 17 microRNAs identified by all four databases were selected for further analysis (Figure 3A). Our previous studies proved that miR-335-5p is down-regulated in NSCLC tissues 20. In addition to miR-335-5p, we found four other up-regulated microRNAs (miR-195-5p, miR-520e, miR-526-5p, and miR-769-3P) in NSCLC tissues, according to the GSE53882 dataset (Figure 3B). GSE43000 was further used to evaluate the differential expression of these selected microRNAs in ADC and SCC tumors in patients with NSCLC. Unfortunately, only miR-195-5p was proven to be down-regulated in SCC compared to ADC tissues (Figure 3C). Kaplan-Meier survival curves further confirmed that patients with higher miR-195-5p expression were associated with more favorable OS (Figure 3D). Furthermore, we validated the result in our additional samples and found that miR-195-5p expression was indeed down-regulated in NSCLC compared to normal tissues (Figure 3E). Interestingly, SCC tumors contained lower miR-195-5p expression compared to the ADC tumors (Figure 3F). All these findings are consistent with the hypothesis that miR-195-5p is one of determinant of the differential CPNE1 expression observed in ADC and SCC tissues.

### MiR-195-5p directly targets CPNE1

**Figure 4:**
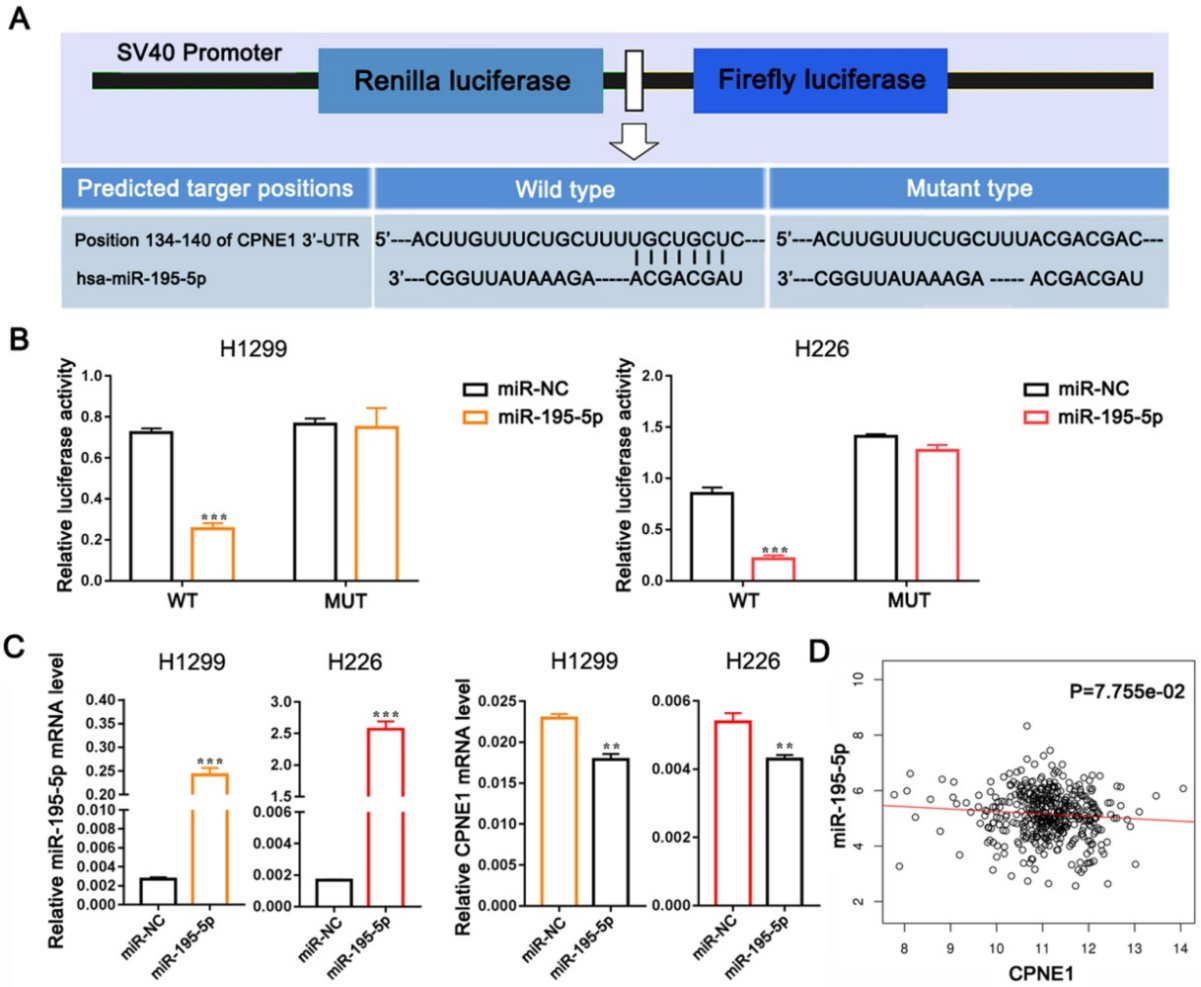
**MiR-195-5p directly targets CPNE1.** (A) Schematic diagram showing the predicted wild-type and mutant miR-195-5p binding site in 3'-UTR of CPNE1. (B) After co-transfection of the constructed plasmids and miR-195-5p, luciferase activity was significantly inhibited in H226 and SK-MES-1 cells. (C) After transfection with miR-195-5p mimics, miR-195-5p expression was elevated and CPNE1 expression was reduced. (D) MiR-195-5p expression was negatively associated with CPNE1 expression. **P<0.01 and ***P<0.001.

To further verify whether CPNE1 was a direct target of miR-195-5p, we subcloned the 3'-UTR of CPNE1 containing the predicted binding site (wild-type), or the corresponding mutated sequence into the psi-CHECK2 vector (Figure 4A). Data clearly showed that miR-195-5p reduced luciferase activity in H1299 and H226 cells transfected with wild-type CPNE1 but not in those transfected with mutant plasmid (Figure 4B). Transfection with miR-195-5p mimics resulted in significant miR-195-5p up-regulation in both cell lines, whereas CPNE1 mRNA was down-regulated (Figure 4C). However, the effect of miR-195-5p on CPNE1 expression did not reach statistical significance and it just showed a trend toward significance based on the Linkedomics public dataset (Figure 4D).

### The function of miR-195-5p in NSCLC cell lines

**Figure 5:**
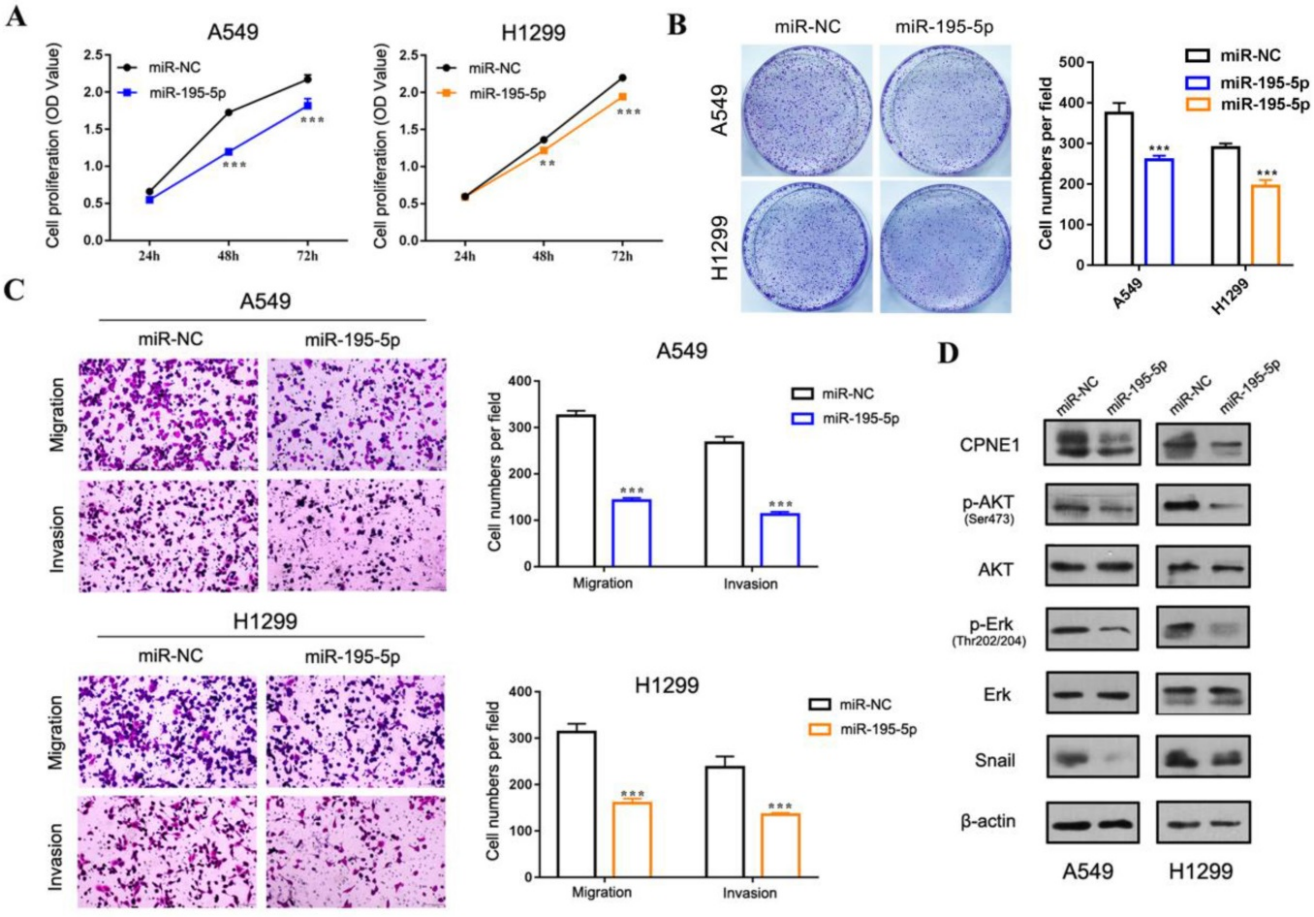
** The function of miR-195-5p in adenocarcinoma cell lines.** (A-B) CCK-8 and clonogenic analysis indicated that miR-195-5p inhibited cell proliferation in A549 and H1299 cells. (C) Transwell assay showed that miR-195-5p inhibited cell migration and invasion (2mm scale bar). (D) Western blot analysis showed that overexpression of miR-195-5p reduced CPNE1 level. Further pathway analysis showed decreased phosphorylation of AKT and Erk under these conditions. The transcription factor Snail was also inhibited. **P<0.01 and ***P<0.001.

**Figure 6:**
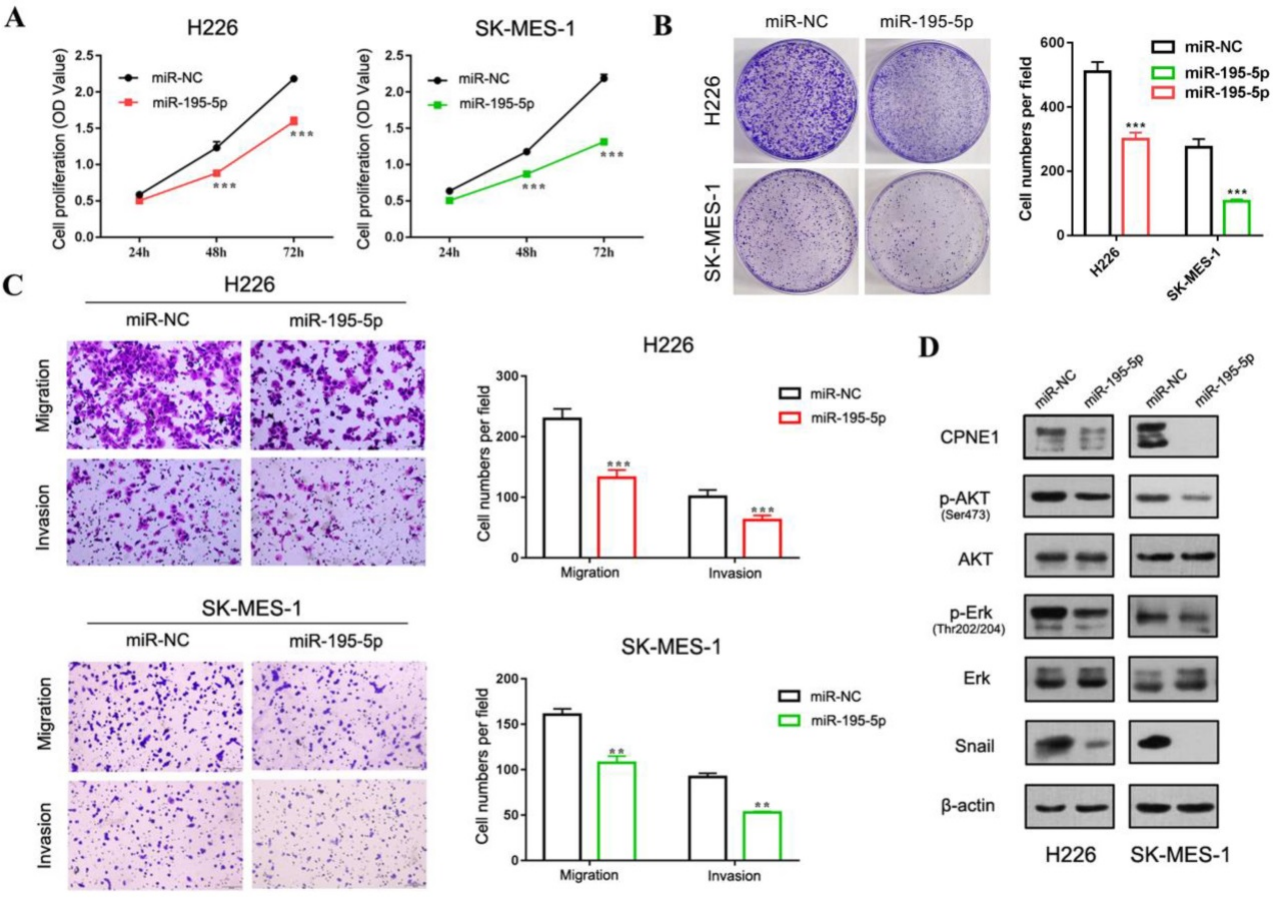
** The function of miR-195-5p in squamous cell lines.** (A-B) CCK-8 and clonogenic analysis indicated that miR-195-5p inhibited cell proliferation in H226 and SK-MES-1 cells. (C) Transwell assay showed that miR-195-5p inhibited cell migration and invasion (2mm scale bar). (D) Western blot analysis showed that the overexpression of miR-195-5p reduced the CPNE1 level. Further pathway analysis showed decreased phosphorylation of AKT and Erk. The transcription factor snail was also inhibited. **P<0.01 and ***P<0.001.

Our previous published paper has demonstrated that CPNE1 functioned as an oncogene in NSCLC cell proliferation, migration and invasion 19, 20. Herein we mainly focus on gaining insight in the role of miR-195-5p in tumorigenesis. To this end, the adenocarcinoma cell lines, A549 and 1299, and the squamous cell lines, H226 and SK-MES-1 were employed. CCK-8 analysis demonstrated decreased NSCLC cell proliferation after transfection with miR-195-5p mimics (Figure 5A and 6A). Similar results were obtained from the clonogenic assay (Figure 5B and 6B). Transwell assay indicated that miR-195-5p overexpression inhibited the migratory and invasive abilities of NSCLC cells (Figure 5C and 6C). Pathway analysis revealed that p-AKT and p-Erk were down-regulated under these conditions. The transcription factor Snail was reported to be involved in epithelial-mesenchymal transition (EMT) process and its high expression contributed to tumor metastasis. In our study, snail was also suppressed in the miR-195-5p transfectants, which was in accordance with the inhibited phenotypes (Figure 5D and 6D).

## Discussion

CPNE1 (Copine I) is an evolutionary conserved protein widely distributed in various tissues and organs 33, 34. Previous studies mainly focused on its role in neuronal differentiation via the participation in intracellular signaling pathways involving AKT, 14-3- 3γ, and JAB1. In addition to its function in the brain, CPNE1 is implicated in tumorigenesis 35. However, its impact on tumor behavior is largely unknown.

CPNE1 was demonstrated to be up-regulated in osteosarcoma and prostate cancer. Additionally, our previous studies have confirmed that CPNE1 is highly overexpressed in NSCLC tissues, compared with non-cancerous lung tissues, and acts as a powerful promoter of NSCLC cell proliferation, migration, and invasion. In our study, we confirmed elevated CPNE1 expression in both our NSCLC tissue samples and the GSE19188 dataset. We also observed that high CPNE1 expression was associated with worse 2-, 5-, and 10-year patient survival. Another public database, Kaplan-Meier Plotter, also attributed prognostic value to CPNE1 up-regulation, which was predictive of worse survival. Different NSCLC subtypes exhibited distinct clinical behaviors and associated genomic alternations, as is the case with differential CPNE1 expression. The oncogenic properties of CPNE1 have been confirmed earlier. However, no data are available regarding possible differences in CPNE1 expression between ADC and SCC tumors. By integrating Oncomine expression data, the Bild and Lee dataset consistently noted that CPNE1 expression is higher in SCC than ADC tumors. Based on GSE19188 data, in the GSE dataset, we reached a similar conclusion. In addition, we divided our patients into subgroups according to the pathological type and found that CPNE1 expression was much lower in ADC than SCC patients. A heat map obtained from the R2 database clearly demonstrated that SCC patients exhibited higher CPNE1 expression and poorer 2-year survival compared to ADC patients.

The reason for CPNE1 differential expression in SCC and ADC tumors has never been explored. MicroRNAs are regarded as single-stranded non-coding RNAs which are well known for their post-transcriptional regulation to control gene expression via directly targeting mRNAs 36. Aberrantly expressed microRNAs in cancer may induce various biological processes, such as cell proliferation, apoptosis, migration, invasion, and metabolism 37, 38. Thus, we hypothesized that microRNA-mRNA interactions accounted for the differential expression of CPNE1 in ADC and SCC tumor subtypes. After integrated bioinformatics analysis, we extracted 17 common dys-regulated microRNAs from four prediction databases, including 12 up-regulated and 5 down-regulated genes. To identify candidate microRNAs involved in CPNE1 differential expression, the GSE4300 dataset was interrogated. Among 5 microRNAs, miR-195-5p was the only one down-regulated in SCC tissues. MiR-195-5p was reported to function as a suppressor in tumor development. In oral squamous cell carcinoma, miR-195-5p can suppress cell proliferation, migration and invasion via targeting TRIM14 39. In ovarian cancer, overexpression of miR-195-5p can reduce cisplatin resistance and angiogenesis via PSAT1-depedent GSK3β/β-catenin signaling pathway 40. To date, only four published papers have demonstrated the suppressive role of miR-195-5p in NSCLC cell proliferation and functioned as a predictor of poor prognosis 28-31. In line with the published result, our further survival analysis proved that relatively low miR-195-5p expression was associated with unfavorable survival. Collectively, miR-195-5p might be regarded as a potential molecular determinant for the differential expression of CPNE1 in ADC and SCC tissues, and may serve as a possible OS indicator in patients.

To further clarify the significance of miR-195-5p in NSCLC pathogenesis, additional *in vitro* functional experiments were performed in both adenocarcinoma and squamous cells. Dual luciferase reporter assay showed that CPNE1 is a direct target of miR-195-5p, which further confirmed our prediction. After transfection with miR-195-5p mimics, CPNE1 expression was down-regulated at mRNA and protein levels. Moreover, CCK-8 analysis showed that cell proliferation was inhibited. Furthermore, the migratory and invasive abilities of four NSCLC cell lines were suppressed, as assessed by transwell assay. Pathway analysis showed that the expression level of p-AKT, p-Erk, and snail was reduced, which most likely accounted for the phenotypic change.

In summary, based on various profile datasets and integrated bioinformatics analysis, we were the first to prove that CPNE1 is more highly expressed in SCC than in ADC tissues. Finally, we identified miR-195-5p as the potential responsible molecular factor. However, due to limited number of samples and technical support, we need to confirm this conclusion in a larger patient cohort.

## Abbreviations

OS: overall survival
ADC: lung adenocarcinoma
SCC: squamous cell lung cancer
NSCLC: non-small cell lung cancer
LCC: large cell cancer
miR-335-5p: microRNA-335-5p
miR-195-5p: microRNA-195-5p
miR-520e: microRNA-520e
miR-526-5p: microRNA-526-5p
miR-769-3p: microRNA-769-3p
3'-UTR: 3'-Untranslated Region
WT: wild type
MUT: mutant type
NC: negative control
CCK-8: cell counting kit-8

## References

[1] Siegel RL, Miller KD, Jemal A (2017). Cancer Statistics, 2017. CA Cancer J Clin.

[2] Li ZY, Luo L, Hu YH (2016). Lung cancer screening: a systematic review of clinical practice guidelines. Int J Clin Pract.

[3] Wood DE, Kazerooni EA, Baum SL (2018). Lung Cancer Screening, Version 3.2018, NCCN Clinical Practice Guidelines in Oncology. J Natl Compr Canc Netw.

[4] Gupta GP, Massague J (2006). Cancer metas tasis: building a framework. Cell.

[5] Reck M, Popat S, Reinmuth N, De Ruysscher D, Kerr KM, Peters S (2014). Metastatic non-small-cell lung cancer (NSCLC): ESMO Clinical Practice Guidelines for diagnosis, treatment and follow-up. Ann Oncol.

[6] Tomsig JL, Creutz CE (2002). Copines: a ubiquitous family of Ca(2+)-dependent phospholipid-binding proteins. Cell Mol Life Sci.

[7] Tomsig JL, Snyder SL, Creutz CE (2003). Identification of targets for calcium signaling through the copine family of proteins. Characterization of a coiled-coil copine-binding motif. J Biol Chem.

[8] Tomsig JL, Creutz CE (2000). Biochemical characterization of copine: a ubiquitous Ca2+-dependent, phospholipid-binding protein. Biochemistry.

[9] Perestenko PV, Pooler AM, Noorbakhshnia M, Gray A, Bauccio C, Jeffrey McIlhinney RA (2010). Copines-1, -2, -3, -6 and -7 show different calcium-dependent intracellular membrane translocation and targeting. FEBS J.

[10] Smith TS, Pineda JM, Donaghy AC, Damer CK (2010). Copine A plays a role in the differentiation of stalk cells and the initiation of culmination in Dictyostelium development. BMC Dev Biol.

[11] Park N, Yoo JC, Lee YS (2014). Copine1 C2 domains have a critical calcium-independent role in the neuronal differentiation of hippocampal progenitor HiB5 cells. Biochem Biophys Res Commun.

[12] Kim TH, Sung SE, Cheal Yoo J (2018). Copine1 regulates neural stem cell functions during brain development. Biochem Biophys Res Commun.

[13] Cheal Yoo J, Park N, Lee B (2017). 14-3-3gamma regulates Copine1-mediated neuronal differentiation in HiB5 hippocampal progenitor cells. Exp Cell Res.

[14] Yoo JC, Park N, Choi HY, Park JY, Yi GS (2018). JAB1 regulates CPNE1-related differentiation via direct binding to CPNE1 in HiB5 hippocampal progenitor cells. Biochem Biophys Res Commun.

[15] Creutz CE, Edwardson JM (2009). Organization and synergistic binding of copine I and annexin A1 on supported lipid bilayers observed by atomic force microscopy. Biochim Biophys Acta.

[16] Ghislat G, Knecht E (2012). New Ca(2+)-dependent regulators of autophagosome maturation. Commun Integr Biol.

[17] Liang J, Zhang J, Ruan J (2017). CPNE1 Is a Useful Prognostic Marker and Is Associated with TNF Receptor-Associated Factor 2 (TRAF2) Expression in Prostate Cancer. Med Sci Monit.

[18] Jiang Z, Jiang J, Zhao B (2018). CPNE1 silencing inhibits the proliferation, invasion and migration of human osteosarcoma cells. Oncol Rep.

[19] Liu S, Tang H, Zhu J (2018). High expression of Copine 1 promotes cell growth and metastasis in human lung adenocarcinoma. Int J Oncol.

[20] Tang H, Zhu J, Du W (2018). CPNE1 is a target of miR-335-5p and plays an important role in the pathogenesis of non-small cell lung cancer. J Exp Clin Cancer Res.

[21] Dawson MA, Kouzarides T (2012). Cancer epigenetics: from mechanism to therapy. Cell.

[22] Costa-Pinheiro P, Montezuma D, Henrique R, Jeronimo C (2015). Diagnostic and prognostic epigenetic biomarkers in cancer. Epigenomics.

[23] Kiselev FL (2014). [MicroRNA and cancer]. Mol Biol (Mosk).

[24] Bartel DP (2004). MicroRNAs: genomics, biogenesis, mechanism, and function. Cell.

[25] Zou J, Liao X, Zhang J (2019). Dysregulation of miR-195-5p/-218-5p/BIRC5 axis predicts a poor prognosis in patients with gastric cancer. J Biol Regul Homeost Agents.

[26] Lin X, Wang S, Sun M (2019). MiR-195-5p/NOTCH2-mediated EMT modulates IL-4 secretion in colorectal cancer to affect M2-like TAM polarization. J Hematol Oncol.

[27] Xu N, Xu J, Zuo Z (2020). Downregulation of lncRNA SNHG12 reversed IGF1R-induced osteosarcoma metastasis and proliferation by targeting miR-195-5p. Gene.

[28] Wang J, Yu X, Ouyang N (2019). MicroRNA and mRNA Interaction Network Regulates the Malignant Transformation of Human Bronchial Epithelial Cells Induced by Cigarette Smoke. Front Oncol.

[29] Li L, Feng T, Zhang W (2020). hsa-miR-195-5pMicroRNA Biomarker for Detecting the Risk of Lung Cancer. Int J Genomics.

[30] Zheng J, Xu T, Chen F, Zhang Y (2019). MiRNA-195-5p Functions as a Tumor Suppressor and a Predictive of Poor Prognosis in Non-small Cell Lung Cancer by Directly Targeting CIAPIN1. Pathol Oncol Res.

[31] Luo J, Pan J, Jin Y (2019). MiR-195-5p Inhibits Proliferation and Induces Apoptosis of Non-Small Cell Lung Cancer Cells by Targeting CEP55. Onco Targets Ther.

[32] Zhu J, Zeng Y, Li W (2017). CD73/NT5E is a target of miR-30a-5p and plays an important role in the pathogenesis of non-small cell lung cancer. Mol Cancer.

[33] Yang W, Ng P, Zhao M, Wong TK, Yiu SM, Lau YL (2008). Promoter-sharing by different genes in human genome-CPNE1 and RBM12 gene pair as an example. BMC Genomics.

[34] Cowland JB, Carter D, Bjerregaard MD, Johnsen AH, Borregaard N, Lollike K (2003). Tissue expression of copines and isolation of copines I and III from the cytosol of human neutrophils. J Leukoc Biol.

[35] Skawran B, Steinemann D, Becker T (2008). Loss of 13q is associated with genes involved in cell cycle and proliferation in dedifferentiated hepatocellular carcinoma. Mod Pathol.

[36] Liu B, Shyr Y, Cai J (2018). Interplay between miRNAs and host genes and their role in cancer. Brief Funct Genomics.

[37] Acunzo M, Croce CM (2015). MicroRNA in Cancer and Cachexia-A Mini-Review. J Infect Dis.

[38] Acunzo M, Romano G, Wernicke D, Croce CM (2015). MicroRNA and cancer-a brief overview. Adv Biol Regul.

[39] International BR (2019). Expression of Concern on "miR-195-5p Suppresses the Proliferation, Migration, and Invasion of Oral Squamous Cell Carcinoma by Targeting TRIM14". Biomed Res Int.

[40] Dai J, Wei R, Zhang P, Kong B (2019). Overexpression of microRNA-195-5p reduces cisplatin resistance and angiogenesis in ovarian cancer by inhibiting the PSAT1-dependent GSK3beta/beta-catenin signaling pathway. J Transl Med.

